# Case Report: *Salmonella Enterica* Serovar Paratyphi B Infection in a Febrile Ill Child during Enhanced Passive Surveillance in an Urban Slum in Mirpur, Dhaka

**DOI:** 10.4269/ajtmh.19-0958

**Published:** 2020-05-26

**Authors:** Farhana Khanam, Nazmul Hasan Rajib, Susan Tonks, Md. Khalequzzaman, Andrew J. Pollard, John D. Clemens, Firdausi Qadri, and the STRATAA study team

**Affiliations:** 1 icddr,b, (International Centre for Diarrhoeal Disease Research, Bangladesh), Dhaka, Bangladesh;; 2 Oxford Vaccine Group, University of Oxford, and the NIHR Oxford Biomedical Research Centre, Oxford, United Kingdom

## Abstract

Paratyphoid fever is one of the major causes of morbidity of febrile illnesses in endemic regions. We report a case of high-grade fever in an infant who was positive for *Salmonella enterica* serovar Paratyphi B (*S*. Paratyphi B) both in blood and stool cultures. The baby was enrolled in the passive surveillance of multicenter, multicomponent epidemiological study of enteric fever (Strategic Typhoid alliance across Africa and Asia; STRATAA) conducted in a population of 110,000 residents over 2 years in an urban slum, Dhaka, Bangladesh. This is the only patient who was positive for *S*. Paratyphi B in blood and stool among more than 6,000 febrile ill patients enrolled in the passive surveillance. The report shows the significance of surveillance to identify changes in the epidemiology of enteric fever.

## INTRODUCTION

Typhoid and paratyphoid fever are collectively known as enteric fever, and the greatest burden of enteric fever is seen among impoverished populations who do not have access to clean drinking water and proper sanitation.^1,2^ *Salmonella enterica* serovar Typhi (*S.* Typhi) and serovar Paratyphi (*S.* Paratyphi A, B, and C) are the etiologies of enteric fever. In recent decades, *S*. Paratyphi A has been the dominant cause of paratyphoid fever in South Asia,^3^ and sporadic reports for *S*. Paratyphi B and C are available.^1,4,5^

A multicenter, multicomponent epidemiological study was carried out to determine the age-stratified burden of enteric fever (Strategic Typhoid alliance across Africa and Asia; STRATAA) conducted in field sites in Bangladesh, Nepal, and Malawi from August 2016 to January 2019.^6^ More than 6,000 febrile ill patients were enrolled in the STRATAA passive surveillance study in Bangladesh in Mirpur, Dhaka, and microbiological culture of blood and stool from patients was carried out to allow assessment of the enteric fever disease burden. A high burden of typhoid and paratyphoid fever was found in both children and adults. In Bangladesh, the overall crude incidence rate of blood culture–confirmed *S.* Typhi was 161 per 100,000 person-years of observation (pyo). For *S.* Paratyphi A, the rates in Bangladesh and Nepal were 117 and 37 per 100,000 pyo in the 5- to 9-year age-group, respectively. In Malawi, there were no cases of *S.* Paratyphi A and *S.* Paratyphi B (unpublished observations). We report here a case of an 11-month-old female infant from whom *S*. Paratyphi B was isolated from both blood and stool at enrollment in a health facility. This is the only case of *S*. Paratyphi B infection for febrile illness surveillance of three study sites.

## CASE PRESENTATION

A febrile female infant, aged 11 months, was brought by her parents to a passive surveillance facility, which was part of the STRATAA surveillance study (Mirpur Field Clinic; MFC) in June 2018 with a history of fever for the past 3 days. The baby was previously healthy. She did not have any significant past medical history, and she was not taking any medication.

Her parents gave a history of vomiting and diarrhea. On examination, the infant was found to be lethargic and irritable, and was crying persistently; she showed signs of some dehydration. The axillary temperature was 39.5°C (103.1°F). There was no evidence of hepatosplenomegaly, and the pulse rate was 110 beats/minutes. The parents of the patient did not give a history of ingestion of street food during the 2 weeks before the onset of symptoms. The mother of the baby explained that they consume boiled water for drinking and use boiled water for preparing food for the infant.

There was no history of antibiotic intake before blood collection. Blood culture was undertaken (BacT/Alert, bioMérieux, Durham, NC) using 3 mL of blood, and stool culture was also performed. Biochemical and serological testing of the bacterial isolates from blood and stool confirmed the diagnosis.^7–9^ *Salmonella enterica* serovar Paratyphi B (*S*. Paratyphi B) was identified, and disk diffusion was used to assess antimicrobial sensitivity using Clinical and Laboratories Standards Institute guidelines.^10^ Strains isolated from blood and stool were both sensitive to all of the tested antibiotics (amoxiclav, ampicillin, cotrimoxazole, ciprofloxacin, chloramphenicol, azithromycin, cefixime, and ceftriaxone). The minimum inhibitory concentration value was determined by the E test for the tested antibiotics (Bio Merieux, Craponne, France). Other laboratory investigation of the patient showed raised C-reactive protein (2.26 mg/dL; reference range: 0.01–0.50 mg/dL).

Treatment of the patient was initiated empirically with azithromycin (10 mg/kg/day) after the collection of blood and stool specimens. Oral rehydration solution, zinc, and paracetamol were also given on the day of enrollment. The patient did not respond for 3 days to treatment and came back to the MFC with a history of high-grade fever. On examination, the axillary temperature was 37.5^°^C (99.5^°^F), and the patient was toxic. Azithromycin treatment was replaced with cefixime (20 mg/kg/day) once the results of the antibiotic susceptibility testing were available. Because the fever had not subsided after 3 days of azithromycin treatment and there is no validated guideline for azithromycin susceptibility for *S*. Paratyphi B,^11^ a switch to cefixime was made empirically at the discretion of the attending physician. The patient was followed up, and physical examination was carried out regularly. No other focus of infection was found. The patient’s condition improved gradually with the treatment of cefixime. Temperature subsided after 9 days of antibiotic treatment, and the patient had fully recovered on day 11 of follow-up.

Follow-up of the patient was carried out at 1 and 6 months following presentation at enrollment, and two stool specimens were collected from the patient, but all cultures were negative. To investigate possible transmission links, stool specimens were collected for microbiological culturing from the household members of the patient.^6^ None of them were positive for *S*. Typhi or for *S*. Paratyphi A/B.

## DISCUSSION

Several studies incorporating existing burden data and modeling approaches at different geographical locations have contributed to the understanding of the global burden of typhoid fever.^3,12–14^ Although few data exist on the global burden of *S*. Paratyphi infection,^1,5,13,15^ *S*. Paratyphi B has an epidemiologic significance and causes outbreaks.^16^

The case report describes the detection of *S*. Paratyphi B infection in one patient who was enrolled in the passive surveillance conducted in a population of 110,000 residents over 2 years in Bangladesh. The patient presented to the healthcare facility with a high-grade fever and was enrolled in the study as a suspected case of typhoid fever, as clinically paratyphoid fever is indistinguishable from typhoid fever and laboratory confirmation is required for accurate diagnosis of the disease.^17,18^ *Salmonella enterica* serovar Paratyphi B was isolated from both blood and stool of the patient. During the acute stage of *Salmonella* infection, patients excrete the organism in their stool, and convalescent and chronic carriers may continue shedding of organism in stool specimens after the resolution of symptoms.^19–24^ However, stool culture was negative in our patient on follow-up at 1 and 6 months after presentation, and negative stool cultures were also obtained from her family members.

The household members of the child patient had no complaints of enteric fever, and their stool culture results did not show any evidence of being asymptomatic carrier of *S*. Paratyphi B. The infant also did not have any history of contact with animals. Hence, the patient could have acquired the infection from the environment while playing in surroundings around the household. Recent studies have suggested that children are more likely to be infected by fecal contamination from the environment in slum settings with contaminated water bodies and open sewers.^25–28^ Improvement of sanitation system will be beneficial for the children who are at risk for acquisition of enteric fever from the environment.^29^

Resistance to first-line antibiotics (chloramphenicol, ampicillin, and cotrimoxazole) has complicated antibiotic choice to treat the patients with typhoid and paratyphoid fever.^30,31^ Third-generation cephalosporins, fluoroquinolones, and azithromycin are widely used to treat enteric fever.^30,31^ Delayed defervescence has been reported for the currently used antibiotics, except fluoroquinolones (ofloxacin and gatifloxacin).^32–34^ Fluoroquinolones are now not recommended for the treatment of enteric fever patients empirically in South Asia as a result of the spread of antimicrobial resistance.^34–36^ Although antibiotic susceptibility testing indicated that the strain isolated from both blood and stool was sensitive to azithromycin, the patient did not respond despite 3 days of treatment. The breakpoints for azithromycin in the treatment of typhoidal *Salmonellae* are uncertain,^37^ and there are few clinical data on response to treatment in *S*. Paratyphi A infection.^38^ In addition, there are no validated guidelines for azithromycin susceptibility in *S*. Paratyphi A/B^37^; hence, further studies are needed to define optimal management for treatment of *S*. Paratyphi B infection. Good responses are expected with the use of third-generation cephalosporin,^34^ and, therefore, the antibiotic was switched to cefixime. However, after initiation of appropriate treatment, it generally takes long time (96–145 hours) for defervescence from enteric fever,^39,40^ as was seen in our patient.

This report shows the presence of *S*. Paratyphi B in South Asia, where *S*. Paratyphi A is considered as the main cause of paratyphoid fever,^41,42^ and highlights the importance of surveillance to identify changes in the epidemiology of enteric fever, particularly in the context of introduction of new typhoid conjugate vaccines to control typhoid, which may promote emergence of paratyphoid as a more significant cause of enteric fever.

## References

[1] Crump JA Mintz ED, 2010 Global trends in typhoid and paratyphoid fever. Clin Infect Dis 50: 241–246. 20014951 10.1086/649541 PMC2798017

[2] Whitaker JA Franco-Paredes C del Rio C Edupuganti S, 2009 Rethinking typhoid fever vaccines: implications for travelers and people living in highly endemic areas. J Travel Med 16: 46–52. 19192128 10.1111/j.1708-8305.2008.00273.x

[3] Crump JA, 2019 Progress in typhoid fever epidemiology. Clin Infect Dis 68 (Suppl 1): S4–S9. 30767000 10.1093/cid/ciy846 PMC6376096

[4] Deen J von Seidlein L Andersen F Elle N White NJ Lubell Y, 2012 Community-acquired bacterial bloodstream infections in developing countries in south and southeast Asia: a systematic review. Lancet Infect Dis 12: 480–487. 22632186 10.1016/S1473-3099(12)70028-2

[5] Buckle GC Walker CL Black RE, 2012 Typhoid fever and paratyphoid fever: systematic review to estimate global morbidity and mortality for 2010. J Global Health 2: 010401. 10.7189/jogh.02.010401 PMC3484760 23198130

[6] Darton TC 2017 The STRATAA study protocol: a programme to assess the burden of enteric fever in Bangladesh, Malawi and Nepal using prospective population census, passive surveillance, serological studies and healthcare utilisation surveys. BMJ Open 7: e016283. 10.1136/bmjopen-2017-016283 PMC5726077 28674145

[7] Islam K Sayeed MA Hossen E Khanam F Charles RC Andrews J Ryan ET Qadri F, 2016 Comparison of the performance of the TPTest, tubex, typhidot and widal immunodiagnostic assays and blood cultures in detecting patients with typhoid fever in Bangladesh, including using a bayesian latent class modeling approach. PLoS Negl Trop Dis 10: e0004558. 27058877 10.1371/journal.pntd.0004558 PMC4825986

[8] Sheikh A 2009 *Salmonella enterica* serovar typhi-specific immunoglobulin A antibody responses in plasma and antibody in lymphocyte supernatant specimens in Bangladeshi patients with suspected typhoid fever. Clin Vaccine Immunol 16: 1587–1594. 19741090 10.1128/CVI.00311-09 PMC2772369

[9] Khanam F 2013 Evaluation of a typhoid/paratyphoid diagnostic assay (TPTest) detecting anti-*Salmonella* IgA in secretions of peripheral blood lymphocytes in patients in Dhaka, Bangladesh. PLoS Negl Trop Dis 7: e2316. 23951368 10.1371/journal.pntd.0002316 PMC3708850

[10] Clinical and Laboratories Standards Institute, 2016 Performance Standards for Antimicrobial Susceptibility Testing; Twenty Sixth Informational Supplement. CLSI Document M100-S26. Wayne, PA: Clinical and Laboratory Standards Institute, 36.

[11] CLSI, 2012 Performance Standards for Antimicrobial Susceptibility Testing: Twenty Second Informational Supplement Ed, CLSI Document M100S-S22. Wayne, PA: CLSI.

[12] Mogasale V Maskery B Ochiai RL Lee JS Mogasale VV Ramani E Kim YE Park JK Wierzba TF, 2014 Burden of typhoid fever in low-income and middle-income countries: a systematic, literature-based update with risk-factor adjustment. Lancet Glob Health 2: e570–e580. 25304633 10.1016/S2214-109X(14)70301-8

[13] Crump JA Luby SP Mintz ED, 2004 The global burden of typhoid fever. Bull World Health Organ 82: 346–353. 15298225 PMC2622843

[14] Lee JS Mogasale VV Mogasale V Lee K, 2016 Geographical distribution of typhoid risk factors in low and middle income countries. BMC Infect Dis 16: 732. 27919235 10.1186/s12879-016-2074-1 PMC5139008

[15] Lozano R 2012 Global and regional mortality from 235 causes of death for 20 age groups in 1990 and 2010: a systematic analysis for the global burden of disease Study 2010. Lancet 380: 2095–2128. 23245604 10.1016/S0140-6736(12)61728-0 PMC10790329

[16] Tasdemir HA Albayrak D, 1989 Antibiotics used for paratyphi B infections resistant to classical treatment and the results of their use. Mikrobiyol Bul 23: 35–39. 2516600

[17] Shimoni Z Pitlik S Leibovici L Samra Z Konigsberger H Drucker M Agmon V Ashkenazi S Weinberger M, 1999 Nontyphoid *Salmonella* bacteremia: age-related differences in clinical presentation, bacteriology, and outcome. Clin Infect Dis 28: 822–827. 10825045 10.1086/515186

[18] Crump JA Youssef FG Luby SP Wasfy MO Rangel JM Taalat M Oun SA Mahoney FJ, 2003 Estimating the incidence of typhoid fever and other febrile illnesses in developing countries. Emerg Infect Dis 9: 539–544. 12737736 10.3201/eid0905.020428 PMC2972755

[19] Vogelsang TM Boe J, 1948 Temporary and chronic carriers of *Salmonella typhi* and *Salmonella* paratyphi B. J Hygiene 46: 252–261. 18122185 10.1017/s0022172400036378 PMC2235125

[20] Im J 2016 Prevalence of *Salmonella* excretion in stool: a community survey in 2 sites, Guinea-Bissau and Senegal. Clin Infect Dis 62 (Suppl 1): S50–S55. 26933022 10.1093/cid/civ789 PMC4772833

[21] Buchwald DS Blaser MJ, 1984 A review of human salmonellosis: II. Duration of excretion following infection with nontyphi *Salmonella*. Rev Infect Dis 6: 345–356. 6377442 10.1093/clinids/6.3.345

[22] Sharp JC, 1970 Convalescent excretion of *Salmonellae* and *Shigellae*. Health Bull 28: 19–22. 5465014

[23] Abboud ES Wahba AH, 1966 Enteric fever carriage with special reference to the incidence of paratyphoid C in Egypt. J Egypt Public Health Assoc 41: 319–327. 4960946

[24] Lackany A Wahdan MH Gaafar MT Mourad AS, 1976 The epidemiology of the carrier state among *enterica* patients in Alexandria. J Egypt Public Health Assoc 51: 233–245. 1025251

[25] Wang LX Li XJ Fang LQ Wang DC Cao WC Kan B, 2012 Association between the incidence of typhoid and paratyphoid fever and meteorological variables in Guizhou, China. Chin Med J 125: 455–460. 22490402

[26] Dewan AM Corner R Hashizume M Ongee ET, 2013 Typhoid fever and its association with environmental factors in the Dhaka metropolitan area of Bangladesh: a spatial and time-series approach. PLoS Negl Trop Dis 7: e1998. 23359825 10.1371/journal.pntd.0001998 PMC3554574

[27] Baker S 2011 Combined high-resolution genotyping and geospatial analysis reveals modes of endemic urban typhoid fever transmission. Open Biol 1: 110008. 22645647 10.1098/rsob.110008 PMC3352080

[28] Sur D Ali M von Seidlein L Manna B Deen JL Acosta CJ Clemens JD Bhattacharya SK, 2007, Comparisons of predictors for typhoid and paratyphoid fever in Kolkata, India. BMC Public Health 7: 289. 17935611 10.1186/1471-2458-7-289 PMC2099435

[29] Akullian A 2015 Environmental transmission of typhoid fever in an urban slum. PLoS Negl Trop Dis 9: e0004212. 26633656 10.1371/journal.pntd.0004212 PMC4669139

[30] Dahiya S 2019 Current antibiotic use in the treatment of enteric fever in children. Indian J Med Res 149: 263–269. 31219092 10.4103/ijmr.IJMR_199_18 PMC6563751

[31] Mishra C Jha A Ahmad M Singh S Ansari A, 2017 A comparative study between cefixime and ofloxacin in the treatment of uncomplicated typhoid fever attending a tertiary care teaching hospital*.* Med Phoenix 2: 3–7.

[32] Sharma P Dahiya S Manral N Kumari B Kumar S Pandey S Sood S Das BK Kapil A, 2018 Changing trends of culture-positive typhoid fever and antimicrobial susceptibility in a tertiary care north Indian hospital over the last decade. Indian J Med Microbiol 36: 70–76. 29735830 10.4103/ijmm.IJMM_17_412

[33] Chandey M Multani AS, 2012 A comparative study of efficacy and safety of azithromycin and ofloxacin in uncomplicated typhoid fever: a randomised, open labelled study. J Clin Diagn Res 6: 1736–1739. 23373040 10.7860/JCDR/2012/4702.2631 PMC3552216

[34] Thompson CN 2017 Treatment response in enteric fever in an era of increasing antimicrobial resistance: an individual patient data analysis of 2092 participants enrolled into 4 randomized, controlled trials in Nepal. Clin Infect Dis 64: 1522–1531. 28329181 10.1093/cid/cix185 PMC5434338

[35] Basnyat B, 2016 Typhoid versus typhus fever in post-earthquake Nepal*.* Lancet Glob Health 4: e516–e517. 27443773 10.1016/S2214-109X(16)30094-8

[36] Wong VK 2015 Phylogeographical analysis of the dominant multidrug-resistant H58 clade of *Salmonella* Typhi identifies inter- and intracontinental transmission events. Nat Genet 47: 632–639. 25961941 10.1038/ng.3281 PMC4921243

[37] CLSI, 2019 Performance Standards/or Antimicrobial Susceptibility Testing, 29th ed Wayne, PA: Clinical and Laboratory Standards Institute, CLSI supplement MlOO.

[38] Parry CM 2015 Clinically and microbiologically derived azithromycin susceptibility breakpoints for *Salmonella enterica* serovars Typhi and paratyphi A. Antimicrob Agents Chemother 59: 2756–2764. 25733500 10.1128/AAC.04729-14 PMC4394775

[39] Dolecek C 2008 A multi-center randomised controlled trial of gatifloxacin versus azithromycin for the treatment of uncomplicated typhoid fever in children and adults in Vietnam. PLoS One 3: e2188. 18493312 10.1371/journal.pone.0002188 PMC2374894

[40] Pandit A 2007 An open randomized comparison of gatifloxacin versus cefixime for the treatment of uncomplicated enteric fever. PLoS One 2: e542. 17593957 10.1371/journal.pone.0000542 PMC1891439

[41] Barkume C 2018 Phase I of the surveillance for enteric fever in Asia project (SEAP): an overview and lessons learned. J Infect Dis 218 (Suppl 4): S188–S194. 30304505 10.1093/infdis/jiy522 PMC6226726

[42] Saha S 2019 Epidemiology of typhoid and paratyphoid: implications for vaccine policy. Clin Infect Dis 68: S117–S123. 30845325 10.1093/cid/ciy1124 PMC6405278

